# Effect of Vitamin D Supplementation on Glycemic Control in Prediabetes: A Meta-Analysis

**DOI:** 10.3390/nu13124464

**Published:** 2021-12-14

**Authors:** Yujing Zhang, Yuan Xue, Dongdong Zhang, Yaping Liu, Ze Xu, Jiaojiao Gao, Wenjie Li, Xing Li

**Affiliations:** College of Public Health, Zhengzhou University, Zhenghzou 450000, China; 15093458874@163.com (Y.Z.); xueyuansnow@163.com (Y.X.); dongdongzhang23@163.com (D.Z.); liuyaping_tang@163.com (Y.L.); ze1995@163.com (Z.X.); gjjatl@163.com (J.G.); lwj@zzu.edu.cn (W.L.)

**Keywords:** vitamin D, prediabetes, glycemic control, meta-analysis

## Abstract

Clinical research results of vitamin D supplementation in the improvement of prediabetes remain controversial. Accordingly, a literature search was conducted of PubMed, Embase (Ovid), and Web of Science prior to 9 November 2021. Randomized controlled studies reported that the following indicators were included: body mass index (BMI), fasting blood glucose (FBG), 2 h oral glucose tolerance test plasma glucose (2h-PG), hemoglobin A1c (HbA1c), insulin resistance by homeostasis model assessment (HOMA-IR), homeostasis model assessment of β-cell function (HOMA-B), and fasting insulin (FINS). Twenty-nine articles (*N* = 3792) were included in the present meta-analysis. Intriguingly, vitamin D supplementation resulted in a vast improvement in FBG (standardized mean difference (SMD) = −0.38; 95%CI: −0.59, −0.16), HbA1c (SMD = −0.14; 95%CI: −0.22, −0.06) and FINS (SMD = 0.18; 95%CI: −0.26, −0.09), but not in other outcomes. However, preferred changes were observed in subgroups, as follows: Asia (SMD_2h-PG_ = −0.25, 95%CI: −0.45, −0.04), study duration ≥1 year (SMD_HOMA-IR_ = −0.44, 95%CI: −0.81, −0.06) (SMD_HOMA-B_ = 0.34, 95%CI: 0.01, 0.66), baseline 25(OH)D < 50 nmol/L (SMD_2h-PG_ = −0.23, 95%CI: −0.39, −0.06), and baseline 25(OH)D ≥ 50 nmol/L (SMD_HOMA-IR_ = −0.50, 95%CI: −0.96, −0.03). In conclusion, oral supplementation of vitamin D has shown better effects in improving FBG, HbA1c, and FINS compared with controls among prediabetics; long-term vitamin D supplementation could have additional effects in participants with vitamin D deficiency for 2h-PG, HOMA-IR, and HOMA-B.

## 1. Introduction

Prediabetes is defined as impaired fasting glucose (IFG) and/or impaired glucose tolerance (IGT), which is characterized by hyperglycemia, insulin resistance, and β-cell dysfunction. It is estimated that the proportion of global IGT in 2019 was 7.5% (374 million) by the International Diabetes Federation (IDF), and 8.6% of adults (548 million) will suffer from IGT by 2045 [1]. Prediabetes is a reversible stage; effective early intervention, such as diet control, exercise enhancement, and drug intervention, can reduce the risk of type 2 diabetes mellitus (T2DM) by 40–70%. Without these interventions, 5–10% of the prediabetic population will develop diabetes every year, and up to 70% will ultimately develop diabetes [2]. Therefore, it is vital to prevent or delay the progression of prediabetes to diabetes.

Vitamin D (VD) is a fat-soluble vitamin that performs its biological functions in the form of vitamin D_2_ and vitamin D_3_. Epidemiological studies have shown that patients with T2DM and prediabetes have lower serum 25(OH)D_3_ levels [3,4]. A four-year follow-up study suggested that low levels of serum 25(OH)D might be linked to the increased incidence of prediabetes or T2DM in Chinese individuals [5]. Two meta-analyses observed that the administration of vitamin D led to a reduction in glucose and hemoglobin A1c (HbA1c), regardless of intramuscular injection and oral supplementation [6,7]. Therefore, more randomized controlled trials (RCTs) are needed to explore the association between vitamin D supplementation and prediabetes.

The aim of the current meta-analysis is to assess the effect of oral vitamin D supplementation on glycemic control in the prediabetic population.

## 2. Materials and Methods

This study was conducted on the basis of the Preferred Reporting Items for Systematic Reviews and Meta-Analysis (PRISMA) statement guidelines.

### 2.1. Literature Search

Articles were searched in the electronic databases of PubMed, Embase (Ovid), and Web of Science from inception to 9 November 2021 using the following search terms: (“vitamin D” OR “vitamin D_2_” OR “vitamin D_3_” OR “alphacalcidol” OR “alfacalcidol” OR “cholecalciferol” OR “ergocalciferol” OR “paricalcitol” OR “doxercalciferol” OR “calcitriol” OR “25-hydroxyvitamin D” OR “25(OH)D”) AND (“prediabetes” OR “pre-diabetes” OR “prediabetic” OR “pre-diabetic” OR “impaired glucose tolerance” OR “impaired fasting glucose” OR “impaired plasma glucose” OR “insulin resistance” OR “hyperglycemia”).

### 2.2. Selection of Studies

The articles were screened and assessed according to our inclusion and exclusion criteria, by two authors independently.

Inclusion criteria:

(1) Population: adults (≥18 years) with prediabetes. Prediabetes criteria [8,9]: (1) IFG: fasting blood glucose (FBG) 6.1–6.9 mmol/L (World Health Organization (WHO)) or 5.6–6.9 mmol/L (American Diabetes Association (ADA)); or (2) IGT: 2h oral glucose tolerance test plasma glucose (2h-PG) 7.8–11.0 mmol/L during an oral glucose tolerance test; or (3) hemoglobin A1c (HbA1c) 5.7–6.4% (ADA).
(2) Intervention: treatment with vitamin D or analogue supplements orally, regardless of the types, dosages, durations, or routines, either with or without calcium (Ca).
(3) Comparison intervention: placebo or nothing.
(4) RCT design.
(5) Eligible studies must have reported at least one of the following coprimary outcomes of interest as defined by the investigators: body mass index (BMI), FBG, 2h-PG, HbA1c, insulin resistance by homeostasis model assessment (HOMA-IR), homeostasis model assessment of β-cell function (HOMA-B), and fasting insulin (FINS). Available sample size, mean and standard deviation (SD) of relevant indexes or data were provided so that mean and SD could be calculated.
(6) Written in English or Chinese.

Exclusion Criteria:

(1) Animal studies, case reports, reviews, or abstracts.
(2) Repeated or overlapped publication.
(3) Trials of participants with diabetes.
(4) Duplicated publications; only the first publication reporting related outcomes was included.

### 2.3. Data Extraction and Risk of Bias Assessment

The following details of each included trial were extracted: last name of the first author, year of publication, country or region, participants, sample size, mean age of the participants, vitamin D type and dose, control group, duration of study, outcome measures.

Two reviewers independently assessed the risk of trial bias based on Cochrane’s risk of bias assessment tool and designated the following items as “high” or “low” or “unclear”: random sequence generation; allocation concealment; blinding of participants and personnel; blinding of outcome assessment; selective reporting; and other sources of bias [10].

### 2.4. Statistical Analysis

An inquiry to authors was set up when SDs were not reported and could not be calculated from the available data. In the absence of data from authors, the SD was estimated from SDs reported by other studies [7].

The effect of vitamin D supplementation on glycemic control was evaluated in the prediabetic population using the standardized mean difference (SMD) and 95% confidence interval (CI). The forest plot was used to visually assess pooled estimates, and it was also used to identify heterogeneity by I^2^ and *p* value [11]; *p* ≤ 0.05 and I^2^ > 50% was considered as heterogeneity. The random-effect model was used to aggregate the results; otherwise, the fixed-effect model was used. To explore the sources of high heterogeneity, subgroup analysis and meta-regression were performed, and sensitivity analysis was used to assess the stability of the results. Subgroup analyses were conducted to illustrate the impacts of certain characteristics: continent (Asia, Europe, and America); vitamin D supplementation (VD vs. placebo, VD + Ca/omage-3 vs. Ca/omage-3, VD+Ca vs. placebo, VD vs. nothing); study duration (≥1 year, <1 year); baseline 25(OH)D (<50 nmol/L, ≥50 nmol/L); biospecimen (serum, plasma, unclear) and HOMA-IR and HOMA-B for different calculating methods (type 1, type 2). The publication bias was defined as “the publication or non-publication of research findings, depending on the nature and direction of the results” in accordance with the Cochrane Handbook, and it was assessed by Begg’s test and funnel plots. Meta-regression was performed to detect published year, continent, vitamin D supplementation, study duration, and baseline 25(OH)D. All the preceding analyses were conducted using Stata 12.0 (Stata, College Station, TX, USA) and Cochrane Review Manager (RevMan) 5.4. The value of *p* < 0.05 was considered statistically significant.

## 3. Results

### 3.1. Literature Search

**Figure 1:**
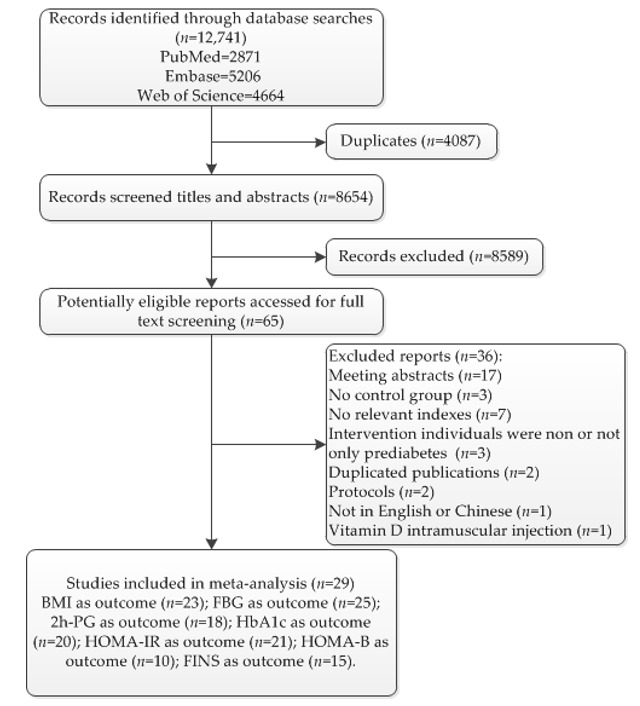
PRISMA flow diagram of the selected studies, indicating the used exclusion criteria. Abbreviations: BMI: body mass index; FBG: fasting blood glucose; 2h-PG: 2 h oral glucose tolerance test plasma glucose; HbA1c: glycosylated hemoglobin A1c; HOMA-IR: insulin resistance by homeostasis model assessment; HOMA-B: homeostasis model assessment of β-cell function; FINS: fasting insulin.

A total of 12,741 potential articles were included after a preliminary search of PubMed, Embase (Ovid), and Web of Science. Among the retrieved articles, 8654 articles were left after removing duplicates. After screening the titles and abstracts, 8589 articles were excluded owing to an irrelevance of the topic. After assessing the full text of the remaining 65 articles, 36 articles were excluded for the following reasons: (1) meeting abstracts (*n* = 17), (2) no control group (*n* = 3), (3) no relevant indexes (*n* = 7), (4) intervention individuals were not or not only prediabetics (*n* = 3), (5) duplicated publications (*n* = 2), (6) protocols (*n* = 2), (7) not in English or Chinese (*n* = 1), and (8) vitamin D intramuscular injection (*n* = 1). A total of 29 articles (*N* = 3792) were included in this meta-analysis (BMI: *n* = 23; FBG: *n* = 25; 2h-PG: *n* = 18; HbA1c: *n* = 20; HOMA-IR: *n* = 21; HOMA-B: *n* = 10; FINS: *n* = 15). The process of screening and selection is shown in Figure 1.

### 3.2. Study Characteristics

Table 1 showed the characteristics of the 29 selected studies in the meta-analysis. All included studies were published between 2007 and 2020 and were carried out in three continents: 12 studies were performed in Asia, 10 studies were conducted in Europe, and seven studies in America. The numbers of participants ranged from 29 to 484, with the mean age ranging from 40 to 76 years old. The intervention periods of vitamin D supplementation were from 8 weeks to 7 years. Twenty-four studies used vitamin D_3_ as the intervention agent, and vitamin D_2_ was used in only one study; moreover, two studies included both vitamin D_3_ and vitamin D_2_ interventions, while the remaining two studies failed to describe the specific type of vitamin D. There were 18 studies that applied vitamin D intervention alone; 10 studies provided vitamin D and calcium co-supplementation (one study involved two intervention groups: vitamin D_3_ and calcium/calcium-placebo co-supplementation); and one study involved vitamin D_3_ and omega-3 co-supplementation or vitamin D_3_ supplementation alone.

### 3.3. Risk of Bias

Figure S1A shows the comprehensive details of the risk of bias assessment. The principal problem detected in the 29 RCTs was that there exists a high risk of bias due to (1) allocation concealment, (2) binding of participants and personnel, and (3) incomplete outcome data. Figure S1B summarizes the proportion of trials with low, unclear, and high risks of bias for each domain. Selection and attrition bias were the primary sources of bias.

### 3.4. Main Analysis

#### 3.4.1. BMI

The change in BMI after vitamin D intervention was reported in 23 studies (*N* = 2854) [12,14,17,18,20,21,22,23,25,26,27,28,29,30,31,32,33,34,35,36,37,39,40]. Random-effects meta-analysis showed no difference in BMI changes between vitamin D supplementation and controls (SMD = 0.01; 95%CI: −0.22, 0.24; *p* = 0.935) (Figure S2), while the results showed statistically significant heterogeneity (I^2^ = 88.7%, *p* < 0.001). Furthermore, subgroup analyses for continent, vitamin D supplementation, baseline 25(OH)D, and duration of the studies were also conducted; the details are presented in Table 2. Only vitamin D + Ca vs. the placebo subgroup exhibited a significant decrease, but conclusions could not be drawn because it only contained one study.

#### 3.4.2. FBG

**Figure 2:**
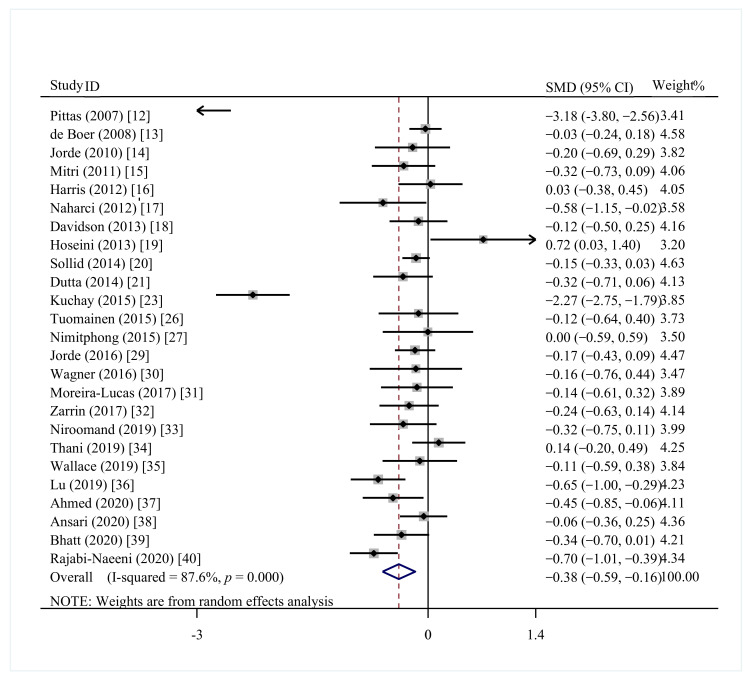
Effect of vitamin D supplementation on fasting blood glucose in prediabetes.

Twenty-five trials involving 3144 participants were included to demonstrate the effect of vitamin D supplementation on FBG in prediabetics [12,13,14,15,16,17,18,19,20,21,23,26,27,29,30,31,32,33,34,35,36,37,38,39,40]. Overall, a significant decrease in FBG content was observed in the vitamin D supplementation group (SMD = −0.38; 95%CI: −0.59, −0.16; *p* = 0.001) (Figure 2), with a high heterogeneity (I^2^ = 87.6%, *p* < 0.001). Subgroup analyses suggested similar findings in the following subgroups: Asia, Europe, vitamin D vs. placebo, study duration ≥ 1 year, study duration <1 year, baseline 25(OH)D < 50 nmol/L, and baseline 25(OH)D ≥ 50 nmol/L (Table 2).

#### 3.4.3. 2h-PG

Changes in 2h-PG were assessed in 18 studies (*N* = 2140) [14,15,16,18,20,21,23,26,27,29,30,31,33,34,35,36,37,39]. Overall, there was no significant difference in 2h-PG reduction between vitamin D supplementation and control groups (SMD = −0.08; 95%CI: −0.22, 0.06; *p* = 0.263) (Figure S3). The heterogeneity was moderate (I^2^ = 56.1%, *p* = 0.002). Nevertheless, effects in the Asia and baseline 25(OH)D < 50 nmol/L subgroups significantly showed a remarkably decrease in the intervention group (Table 2).

#### 3.4.4. HbA1c

**Figure 3:**
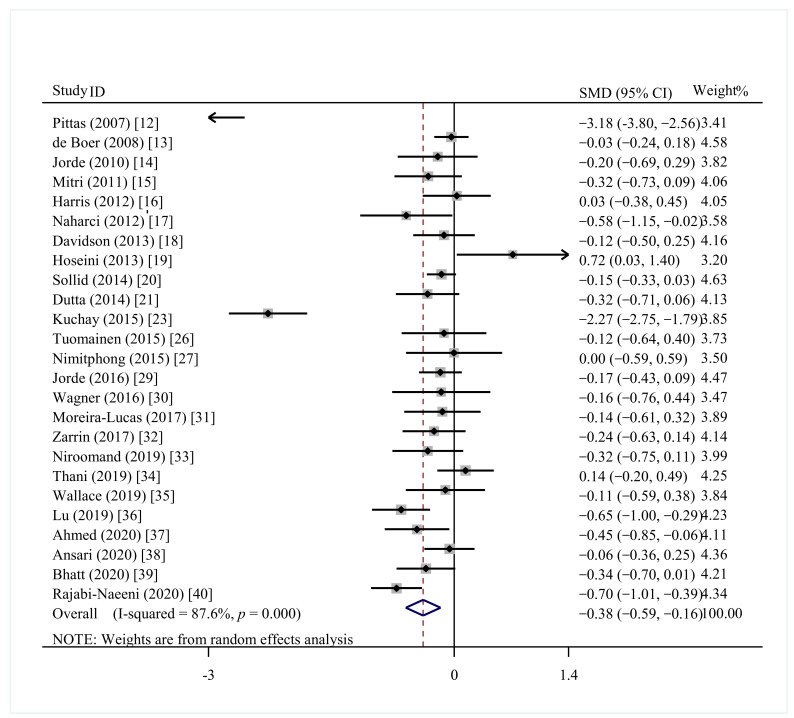
Effect of vitamin D supplementation on glycosylated hemoglobin A1c in prediabetes.

The effect of vitamin D supplementation on HbA1c was illustrated in 20 studies (*N* = 2470) [14,15,16,18,19,20,21,23,24,25,26,27,28,29,30,31,32,34,35,37]. A larger reduction in HbA1c content was found in prediabetes with vitamin D supplementation than in controls (SMD = −0.14; 95%CI: −0.22, −0.06; *p* = 0.001) (Figure 3), although there was a moderate heterogeneity (I^2^ = 46.5%, *p* = 0.012). The effects shown in Asia, America, vitamin D vs. placebo, vitamin D vs. nothing, study duration ≥ 1 year, and baseline 25(OH)D < 50 nmol/L participants were significantly in favor of the vitamin D supplementation (Table 2).

#### 3.4.5. HOMA-IR

There were 21 (*N* = 2748) out of 29 RCTs that reported HOMA-IR [12,13,14,16,17,18,19,20,21,22,26,27,29,32,33,34,35,36,37,39,40]. Results showed that vitamin D supplementation had no significant impact on HOMA-IR (SMD = −0.15; 95%CI: −0.50, 0.20; *p* = 0.402) (Figure S4), with the heterogeneity relatively high (I^2^ = 94.8%, *p* < 0.001). However, the following subgroups favor the vitamin D supplementation group: study duration ≥ 1 year and baseline 25(OH)D ≥ 50 nmol/L (Table 2).

#### 3.4.6. HOMA-B

The results of 10 clinical trials (*N* = 928) revealed that treatment with vitamin D failed to restore HOMA-B (SMD = 0.19; 95%CI: −0.09, 0.47; *p* = 0.179); the results showed high heterogeneity (I^2^ = 75.9%, *p* < 0.001) (Figure S5) [18,19,21,26,27,31,34,35,36,40]. The subgroup of study duration ≥1 year was significantly in favor of vitamin D supplementation (Table 2).

#### 3.4.7. FINS

**Figure 4:**
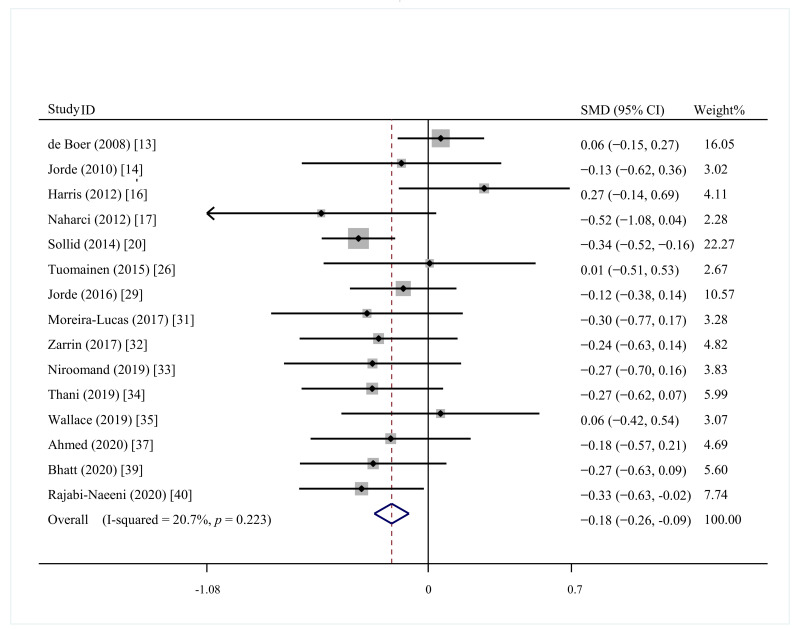
Effect of vitamin D supplementation on fasting serum insulin in prediabetes.

Fourteen trials encompassing 2172 participants were included [13,16,17,20,26,29,31,32,33,34,35,37,39,40]. The present meta-analysis found that vitamin D supplementation had a beneficial effect on FINS in prediabetics (SMD = 0.18; 95%CI: −0.26, −0.09; *p* < 0.001, with low heterogeneity (I^2^ = 20.7%, *p* = 0.223) (Figure 4). The subgroup of Asia, Europe, vitamin D vs. placebo, study duration ≥ 1 year, study duration < 1 year, and baseline 25(OH)D < 50 nmol/L showed similar results (Table 2).

### 3.5. Meta-Regression

To uncover the potential causes of high heterogeneity, a meta-regression analysis was performed involving publication year, geographic locations, vitamin D supplementation, study duration, and baseline 25(OH)D. None were found to contribute to the heterogeneity between studies.

### 3.6. Sensitivity Analysis

In the sensitivity analysis, the results of BMI, 2h-PG, and HOMA-B levels remained basically robust. The remarkable effect of vitamin D supplementation on HbA1c disappeared when excluding Kuchey et al. [23], and a similar trend was found for FINS when excluding de Boer et al. [13] and Sollid et al. [20], as the heterogeneity decreased to 0.00%. However, the effect of vitamin D on HOMA-IR in prediabetes turned to significant (SMD = −0.23; 95%CI: −0.42, −0.03; *p* < 0.001), with high heterogeneity (I^2^ = 82.5%, *p* < 0.001), when excluding Pittas et al. [12] and Bhatt et al. [39]. In addition, by excluding Pittas et al. [12] and Kuchey et al. [23] from the meta-analysis, the heterogeneity for the beneficial effect of vitamin D supplementation on FBG decreased from 87.6% to 41.5%.

### 3.7. Publication Bias

**Figure 5:**
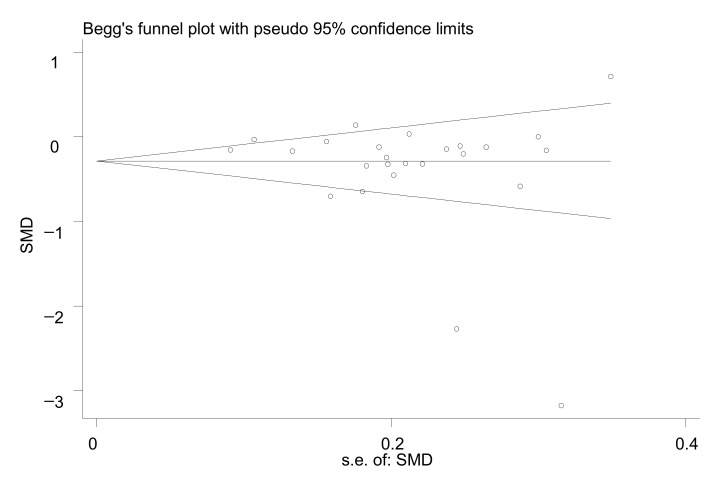
Funnel plot of fasting blood glucose changes.

The funnel plot showed that the study distribution was basically symmetrical (Figure 5), and statistical tests showed no evidence of a publication bias (Begg’s test: *p* = 0.151, 95%CI: −5.76, 0.94).

## 4. Discussion

This meta-analysis showed a positive impact of vitamin D supplementation on FBG, HbA1c, and FINS in prediabetes but showed no benefit of vitamin D supplementation with regard to improving BMI, 2h-PG, HOMA-IR, and HOMA-B.

The results of the present research are consistent with Poolsup et al. [7] in terms of FBG, 2h-PG, HbA1c, and HOMA-IR, while the results for HOMA-IR are partially consistent with Yu et al. [6]. The present analysis contained the most comprehensive review of trials available compared with previous meta-analyses [6,7]. In addition to the indicators reported in previous studies (e.g., FBG, 2h-PG, HbA1c, HOMA-IR), we added the following indexes to better reflect the control of FBG and the protection of islets in prediabetes by vitamin D supplementation: HOMA-B, FINS, and BMI.

We found that vitamin D supplementation generated various effects among different continents: significant reductions in FBG, 2h-PG, HbA1c, and FINS levels were seen in Asian populations, FBG and FINS reductions were found in Europeans, and decreased HbA1c was detected in Americans, with the same index falling even more sharply in Asians. Asians showed a higher level of free 25(OH)D than the Europeans and Americans after the same dose of vitamin D_3_ supplementation, which may reflect the discrepancy in the affinity of vitamin D binding protein to 25(OH)D [41]. In addition to vitamin D binding protein, cytochrome P450 family 2 subfamily R polypeptide 1 (*CYP2R1*) and vitamin D receptor (*VDR*) also affected the vitamin D status, metabolism, and function. Jones et al. [42] reported that the frequencies of *CYP2R1* and *VDR* variants differed by geographic areas. Elkum et al. [43] found *CYP2R1* genes associated with vitamin D status in Arabs (*CYP2R1* rs10741657, rs10500804 and rs12794714) and South Asians (*CYP2R1* rs10741657) but not Southeast Asians. Lu et al. [44] confirmed that *CYP2R1* rs2060793 variant was associated with serum 25(OH)D levels in the Shanghai but not the Beijing subpopulation. The *VDR* were similar to *CYP2R1* genes, showing differences between races [45,46,47]. In addition, Rajan et al. and Weishaar et al. confirmed that people with darker skin color (Asians’ skin was noted as darker than Europeans and Americans) were independently and significantly associated with poor vitamin D status, which may be related to better FBG improvement in Asians [48,49].

The results of the duration of the intervention were inconsistent with previous studies [7]. Perhaps due to the small sample size, the meta-analysis of Poolsup et al. did not reached a conclusion in relation to study duration and vitamin D supplementation effect. This analysis included 19 additional studies and revealed that interventions of vitamin D ≥ 1 year were able to improve the contents of FBG, HbA1c, HOMA-IR, HOMA-B, and FINS in prediabetes; FBG and FINS in patients with prediabetes were also improved when intervention durations were < 1 year. Previous studies [50,51] found that the protective effects of vitamin D supplementation on T2DM were primarily generated by intervention periods of less than three months. The reason for this was not only the difference in dosage; the progression of diabetes could also weaken the effect of long-term intervention over time. Prediabetes is a reversible process, but T2DM is not [52]. Therefore, the longer the vitamin D supplementation in prediabetes, the more effective the prevention of the onset of diabetes and the less likely the disease will progress.

A cross-sectional analysis conducted by the U.S. National Health and Nutrition Examination Survey (2007–2012) found that serum 25(OH)D levels from deficient (<50 nmol/L) or insufficient (50.1–74.9 nmol/L) individuals were more likely to suffer from prediabetes compared to sufficient individuals (>75 nmol/L) [53]. In addition, studies have also shown that 25(OH)D levels are inversely associated with the prevalence of diabetes and HOMA-IR [54,55]. Subgroup analysis related to baseline 25(OH)D levels showed that, in subjects with baseline 25(OH)D < 50 nmol/L, treatment of vitamin D significantly improved FBG, 2h-PG, HbA1c, and FINS; FBG showed a better improvement effect among subjects with baseline 25(OH)D ≥ 50 nmol/L. In addition, there was also a notably ameliorate effect on HOMA-IR. Indicators of improvement were not entirely consistent in participants with different baseline 25(OH)D levels; a possible reason for this is that vitamin D could directly act on the receptors on the pancreas to improve islet function, while the mechanism of insulin resistance is more complicated and requires longer intervention to achieve the effect [56].

First, to our knowledge, this meta-analysis is unique, as more indicators were used to elaborate the effects of vitamin D supplementation on prediabetes. Second, the Cochrane Collaboration tool was used to evaluate the quality of the trials. Third, we explored influential factors, including continents, intervention strategies, intervention durations, baseline 25(OH)D, and different calculating methods (specifically applicable to HOMA-IR and HOMA-B). Lastly, only RCTs with oral vitamin D supplementation were included in the meta-analysis. However, the heterogeneity of the results was still significant because of discrepancies in vitamin D types, doses, study durations, participants, and some unknown factors. Other limitations were that most of containing studies did not evaluate the effects of sun exposure, dietary intake, and physical exercise, as different lifestyles and genes between studies may affect vitamin status [57,58,59]. Third, the intervention and control groups were unbalanced; some control groups from different studies were without a matching placebo [17,19,21,23,27,36,38], and participants clearly knowing their groupings might result in a Hawthorne effect. Fourth, the biospecimens were nearly all serum and plasma samples (some studies were unclear); subgroup analyses revealed that glucose indexes were not affected by the difference in biospecimens, but there were three studies on FINS that were collected either from plasma or otherwise. The specific reason needs to be studied due to the small number of studies. Finally, some studies included two intervention groups; some intervention participants underwent dose changes [17,19,21,23,26,27,28,33,38], and we were unable to accurately determine the relationship between individual doses and improvement in indicators.

## 5. Conclusions

Oral supplementation of vitamin D has been shown to exert better effects in improving FBG, HbA1c, and FINS compared with controls among prediabetes; it has not been found to regulate BMI. Long-term vitamin D supplementation could show an additional effect in participants with vitamin D deficiency for 2h-PG, HOMA-IR, and HOMA-B. More large-scaled clinical trials are needed to elucidate the association between vitamin D and prediabetes.

## Figures and Tables

**Table 1:** Characteristics of studies included in the meta-analysis.

Study	Year	Country	Participants	*N*(I/C)	Age (Years)	Intervention	Control	Duration Period	Outcomes
Pittas et al. [12]	2007	USA	IFG	45/47	I: 71.1 ± 0.7 C: 71.3 ± 0.8	VD_3_ 700 IU/day + Ca 500 mg/day	placebo	3 years	①②⑤
de Boer et al. [13]	2008	USA	IFG	172/178	NA	VD_3_ 400 IU/day + Ca 1000 mg/day	placebo	7 years	②⑤⑦
Jorde et al. [14]	2010	Norway	Prediabetes	34/31	NA	VD_3_ 20,000 or 40,000 IU/week + Ca 500 mg/day	placebo + Ca 500 mg/day	1 year	①②③④⑤⑦
Mitri et al. [15]	2011	USA	Prediabetes	46/46	I: 57 ± 9.59 C: 58 ± 9.60	VD_3_ 2000 IU/day + Ca 800 mg/day or Ca-placebo	VD-placebo + Ca 800 mg/day or Ca-placebo	16 weeks	②③④
Harris et al. [16]	2012	USA	Prediabetes	43/46	I: 71.1 ± 0.7 C: 56.3 ± 12.3	VD_3_ 4000 IU/day + Ca 600 mg/day	placebo + Ca 600 mg/day	12 weeks	②③④⑤⑦
Naharci et al. [17]	2012	Turkey	IFG	28/23	I: 75.1 ± 7.3 C: 76.1 ± 5.4	VD insufficient: VD_3_ 800 IU/day + Ca 1000 mg/day VD normal: VD_3_ 400 IU/day + Ca 600 mg/day	nothing	4.7 ± 2.5 months	①②⑤⑦
Davidson et al. [18]	2013	USA	Prediabetes	56/53	NA	VD_3_ 88,865 IU/week (range 64,731–13,4446 IU/week)	placebo	1 year	①②③④⑤⑥
Hoseini et al. [19]	2013	Iran	Prediabetes	21/15	I: 46.3 ± 6.5 C: 48.9 ± 6.1	VD 50,000 IU/week or every other week + Ca 500 mg/day	Ca 500 mg/day	12 weeks	②④⑤⑥
Sollid et al. [20]	2014	Norway	Prediabetes	242/242	NA	VD_3_ 20,000 IU/week	placebo	1 years	①②③④⑤⑦
Dutta et al. [21]	2014	India	Prediabetes	55/49	NA	VD_3_ 60,000 IU/week for 8 weeks, then 60,000 IU/month + Ca 500 mg/day	Ca 500 mg/day	I: 28.2 ± 8.83 months C: 29.15 ± 7.69 months	①②③④⑤⑥
Oosterwerff et al. [22]	2014	Netherlands	Prediabetes	65/65	NA	VD_3_ 1200 IU/day + Ca 500	placebo + Ca 500 mg/day	16 months	①⑤
						mg/day			
Kuchay et al. [23]	2015	India	Prediabetes	56/55	NA	VD_3_ 60,000 IU/week for 4 weeks and then 60,000 IU/month	nothing	1 year	①②③④
Barengolts et al. [24]	2015	USA	Prediabetes	87/86	I: 58.2 ± 6.0 C: 59.8 ± 6.0	VD_2_ 50,000 IU/week	placebo	1 year	④
Didriksen et al. [25]	2015	Norway	IFG	18/11	I: 60.8 ± 9.33 C: 62.0 ± 9.41	VD_3_ 20,000 IU/week	placebo	3–5 years	①④
Tuomainen et al. [26]	2015	Finland	Prediabetes	45/21	NA	VD_3_ 1600/3200 IU/day	placebo	5 months	①②③④⑤⑥⑦
Nimitphong et al. [27]	2015	Thailand	Prediabetes	29/18	I: 61.8 ± 9.7 C: 57.9 ± 13.3	VD_2_ 20,000 IU/week or VD_3_ 15,000 IU/week	nothing	3 months	①②③④⑤⑥
Forouhi et al. [28]	2016	UK	Prediabetes	210/111	NA	VD_2_ 100,000 IU/month or VD_3_ 100,000 IU/month	placebo	4 months	①④
Jorde et al. [29]	2016	Norway	Prediabetes	116/111	NA	VD_3_ 20,000 IU/week	placebo	5 years	①②③④⑤⑦
Wagner et al. [30]	2016	Sweden	Prediabetes	21/22	I:66.52 ± 4.29 C: 66.71 ± 3.01	VD_3_ 30,000 IU/week	placebo	8 weeks	①②③④
Moreira-Lucas et al. [31]	2017	Canada	IFG	35/36	I: 49.1 ± 13.9 C: 45.6 ± 14.3	VD_3_ 28,000 IU/week	placebo	24 weeks	①②③④⑥⑦
Zarrin et al. [32]	2017	Iran	Prediabetes	51/53	I: 48.11 ± 7.6 C: 48.43 ± 7.7	VD_3_ 1000 IU/day	placebo	3 months	①②④⑤⑦
Niroomand et al. [33]	2019	Iran	Prediabetes	43/40	NA	VD_3_ 50,000 IU/week for 3 months and then 50,000 IU/month	placebo	6 months	①②③⑤⑦
Thani et al. [34]	2019	Qatar	Prediabetes	57/75	I: 45.51 ± 8.96 C: 44.89 ± 8.88	VD_3_ 30,000 IU/week	placebo	6 months	①②③④⑤⑥⑦
Wallace et al. [35]	2019	UK	Prediabetes	35/31	I: 52.4 ± 2.0 C: 54.0 ± 1.7	VD_3_ 3000 IU/day	placebo	26 weeks	①②③④⑤⑥⑦
Lu et al. [36]	2019	China	Prediabetes	64/65	I: 59.29 ± 6.07 C: 59.41 ± 9.10	VD 400 IU/day	nothing	1 year	①②③⑤⑥
Ahmed et al. [37]	2020	India	Prediabetes	52/49	I: 41.1 ± 8 C: 41.6 ± 7	VD_3_ 60,000 IU/week	placebo	12 weeks	①②③④⑤⑦
Ansari et al. [38]	2020	Italy	Prediabetes	146/57	NA	VD_3_ 50,000 IU/week for 2 months, then twice a month for the next 2 months, followed by 1000 IU/day for the last 2 months	nothing	6 months	②
Bhatt et al. [39]	2020	India	Prediabetes	61/60	NA	VD_3_ 60,000 IU/week + CaCO_3_ 1 gm/day	placebo + CaCO_3_ 1 gm/day	78 weeks	①②③⑤⑦
Rajabi-Naeeni et al. [40]	2020	Iran	Prediabetes	84/84	I: 39.46 ± 6.91 C: 40.82 ± 7.19	VD_3_ 60,000 IU/2 weeks + omega-3 1000 mg twice/day or not	omega-3 1000 mg twice/day or omega-3 placebo + VD_3_ placebo	8 weeks	①②⑤⑥⑦

Abbreviations: N: number of participants; I: intervention group; C: control group; IU: international units; IFG: impaired fasting glucose; VD: vitamin D; Ca: calcium. ① body mass index; ② fasting blood glucose; ③ 2 h oral glucose tolerance test plasma glucose; ④ glycosylated hemoglobin A1c; ⑤ insulin resistance by homeostasis model assessment; ⑥homeostasis model assessment of β-cell function; ⑦ fasting serum insulin. Partial data of age and duration period were expressed as mean±standard deviation.

**Table 2:** Subgroup analyses.

Subgroup	BMI			FBG				2h-PG				HbA1c			
	*n*	SMD (95%CI)	*p*	*n*	SMD (95%CI)		*p*	*n*	SMD (95%CI)		*p*	*n*	SMD (95%CI)	*p*	
Continent															
Asia	11	0.06 (−0.38, 0.49)	0.796	12	−0.43 (−0.77, −0.10)		0.012	8	−0.25 (−0.45, −0.04)		0.019	7	−0.30 (−0.45, −0.14)	0.007	
Europe	9	0.04 (−0.28, 0.35)	0.814	7	−0.14 (−0.26, −0.02)		0.020	6	0.13 (−0.09, 0.35)		0.252	8	−0.04 (−0.15, 0.07)	0.478	
America	3	−0.25 (−0.59, 0.09)	0.146	6	−0.59 (−1.26, 0.08)		0.083	4	−0.06 (−0.27, 0.15)		0.577	5	−0.20 (−0.37, −0.03)	0.020	
VD supplementation															
VD vs. placebo	13	−0.09 (−0.28, 0.09)	0.317	11	−0.16 (−0.26, −0.06)		0.002	10	−0.04 (−0.19, 0.11)		0.618	13	−0.10 (−0.19, −0.01)	0.035	
VD + Ca/omage-3 vs. Ca/omage-3	6	−0.07 (−0.64, 0.50)	0.809	7	−0.23 (−0.5, 0.04)		0.098	5	−0.05 (−0.32, 0.23)		0.744	5	−0.19 (−0.39, 0.01)	0.069	
VD + Ca vs. placebo	1	−0.59 (−1.00, −0.17)	0.006	2	−1.59 (−4.67, 1.49)		0.312	—	—		—	—	—	—	
VD vs. nothing	3	0.81 (−0.27, 1.90)	0.142	5	−0.71 (−1.48, 0.06)		0.069	3	−0.22 (−0.77, 0.32)		0.420	2	−0.60 (−0.93, −0.27)	<0.001	
Duration															
≥1 year	13	0.10 (−0.25, 0.45)	0.571	15	−0.51 (−0.85, −0.18)		0.003	11	−0.11 (−0.28, 0.06)		0.208	13	−0.19 (−0.29, −0.09)	<0.001	
<1 year	10	−0.12 (−0.40, 0.17)	0.420	10	−0.16 (−0.28, −0.04)		0.010	7	−0.02 (−0.28, 0.24)		0.882	7	−0.06 (−0.19, 0.07)	0.348	
Baseline 25 (OH)D															
<50 nmol/L	12	0.00 (−0.35, 0.36)	0.980	13	−0.36 (−0.66, −0.07)		0.017	10	−0.23 (−0.39, −0.06)		0.007	10	−0.22 (−0.34, −0.09)	0.001	
≥50 nmol/L	10	−0.16 (−0.36, 0.04)	0.120	10	−0.41 (−0.81, −0.01)		0.044	7	0.13 (−0.07, 0.33)		0.213	10	−0.09 (−0.19, 0.01)	0.089	
Type															
1	—	—	—	—	—		—	—	—		—	—	—	—	
2	—	—	—	—	—		—	—	—		—	—	—	—	
Biospecimen															
Serum	—	—	—	5	−0.42 (−0.67, −0.16)		0.002	3	−0.01 (−0.15, 0.12)		0.849	—	—	—	
Plasma	—	—	—	15	−0.43 (−0.85, −0.01)		0.044	13	−0.06 (−0.27, 0.15)		0.598	—	—	—	
Unclear	—	—	—	5	−0.14 (−0.28, −0.01)		0.038	2	−0.20 (−0.47, 0.06)		0.128	—	—	—	
**Subgroup**	**HOMA-IR**				**HOMA-B**					**FINS**					
	***n***	**SMD (95%CI)**	***p***		***n***	**SMD (95%CI)**			***p***	***n***	**SMD (95%CI)**				***p***
Continent															
Asia	11	0.08 (−0.55, 0.72)	0.799		6	0.22 (−0.23, 0.67)			0.347	7	−0.28 (−0.43,−0.14)				<0.001
Europe	6	−0.17 (−0.55, 0.20)	0.361		2	0.20 (−0.16, 0.55)			0.278	5	−0.22 (−0.35, −0.09)				0.001
America	4	−0.75 (−1.84, 0.33)	0.172		2	0.20 (−0.09, 0.50)			0.175	3	0.05 (−0.13, 0.22)				0.584
VD supplementation															
VD vs. placebo	9	−0.23 (−0.46, 0.00)	0.054		5	0.05 (−0.24, 0.34)			0.718	9	−0.23 (−0.34, −0.13)				<0.001
VD + Ca/omage-3 vs. Ca/omage-3	7	0.60 (−0.28, 1.49)	0.181		3	0.39 (−0.32, 1.10)			0.286	4	−0.16 (−0.35, 0.03)				0.093
VD + Ca vs. placebo	2	−1.62 (−4.94, 1.69)	0.337		—	—			—	1	0.06 (−0.15, 0.27)				0.564
VD vs. nothing	3	−0.75 (−1.67, 0.17)	0.110		2	0.31 (−0.57, 1.18)			0.492	1	−0.52 (−1.08, 0.04)				0.069
Duration															
≥1 year	13	−0.44 (−0.81, −0.06)	0.023		7	0.34 (0.01, 0.66)			0.041	8	−0.16 (−0.26, −0.05)				0.004
<1 year	8	0.35 (−0.42, 1.12)	0.374		3	−0.23 (−0.49, 0.03)			0.084	7	−0.22 (−0.36, −0.08)				0.003
Baseline 25 (OH)D															
<50 nmol/L	10	0.29 (−0.31, 0.89)	0.346		4	−0.06 (−0.34, 0.23)			0.688	9	−0.19 (−0.33, −0.05)				0.008
≥50 nmol/L	9	−0.50 (−0.96, −0.03)	0.035		5	0.27 (−0.16, 0.71)			0.214	5	−0.25 (−0.38, −0.13)				<0.001
Type															
1	18	−0.16 (−0.56, 0.24)	0.441		6	0.35 (−0.08, 0.78)			0.109	—	—				—
2	3	−0.13 (−0.41, 0.14)	0.335		4	−0.03 (−0.27, 0.20)			0.795	—	—				—
Biospecimen															
Serum	—	—	—		—	—			—	12	−0.23 (−0.33, −0.13)				<0.001
Plasma	—	—	—		—	—			—	2	−0.19 (−0.47, 0.10)				0.205
Unclear	—	—	—		—	—			—	1	0.06 (−0.15, 0.27)				0.564

Abbreviations: VD: vitamin D; BMI: body mass index; FBG: fasting blood glucose; 2h-PG: 2 h oral glucose tolerance test plasma glucose; HbA1c: hemoglobin A1c; HOMA-IR: insulin resistance by homeostasis model assessment; HOMA-B: homeostasis model assessment of β-cell function; FINS: fasting insulin.

## Data Availability

All data relevant to the study are included in the article.

## Supplementary Materials

The following are available online at https://www.mdpi.com/article/10.3390/nu13124464/s1, Figure S1: Risk of bias assessment of the included studies using the Cochrane Collaboration tool (A) Risk of bias in included studies; (B) Risk of bias across each bias domain, Figure S2: Effect of vitamin D supplementation on body mass index in prediabetes, Figure S3: Effect of vitamin D supplementation on 2h oral glucose tolerance test plasma glucose in prediabetes, Figure S4: Effect of vitamin D supplementation on insulin resistance by homeostasis model assessment in prediabetes, Figure S5: Effect of vitamin D supplementation on homeostasis model assessment of β-cell function in prediabetes.
