# Supplementary material for: Effect of Vitamin D Supplementation on Glycemic Control in Prediabetes: A Meta-Analysis
Source: Nutrients. 2021 Dec 14;13(12):4464. doi: 10.3390/nu13124464 (PMC8707376; doi:10.3390/nu13124464)
Supplement: Supplementary file 1 [file nutrients-13-04464-s001.zip › nutrients-1480285-supplementary.pdf]

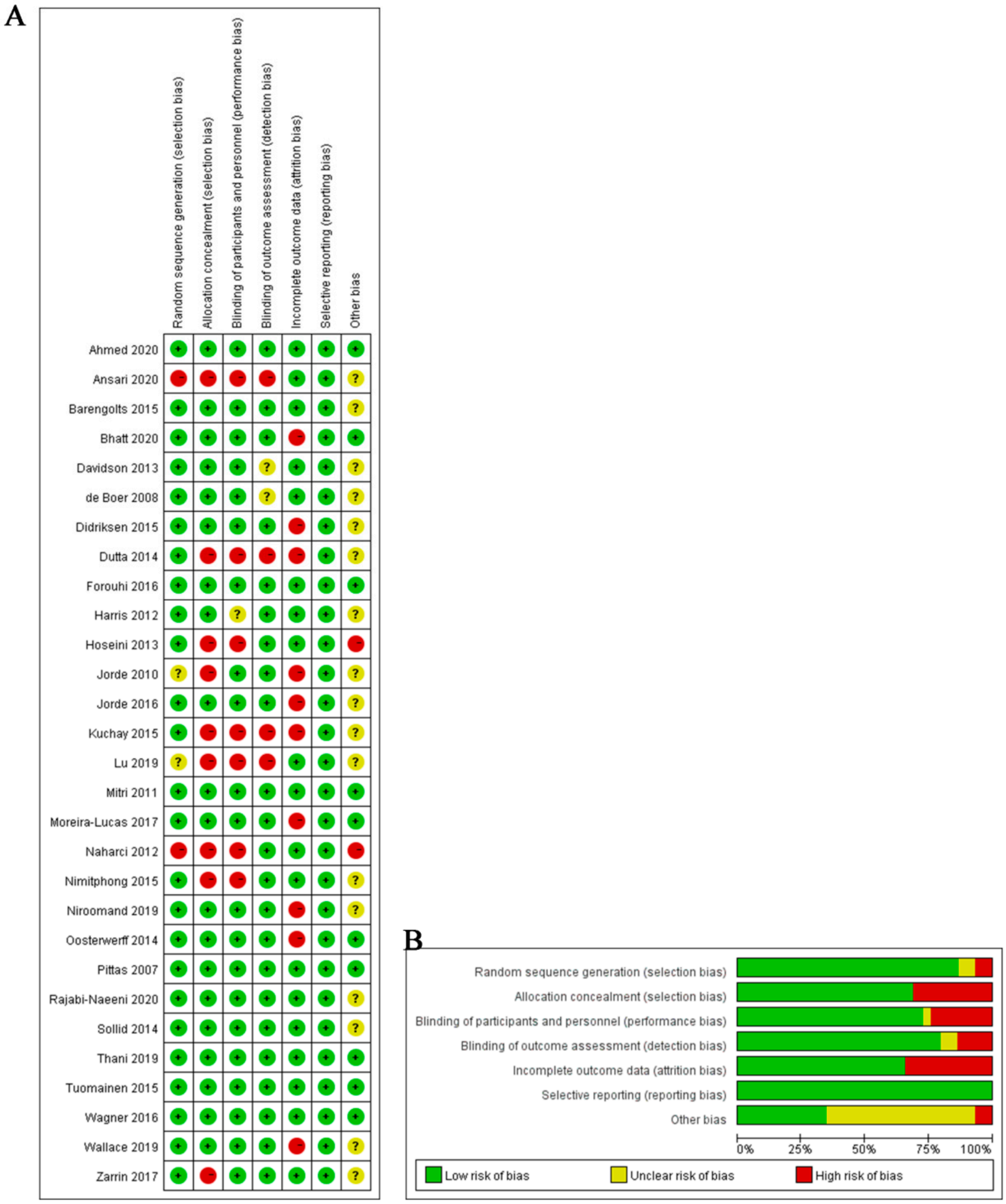

Figure S1. Risk of bias assessment of the included studies using the Cochrane Collaboration tool. (A) Risk of bias in included studies; (B) Risk of bias across each bias domain. Risk of bias levels: green (low), yellow (unclear), red (high).

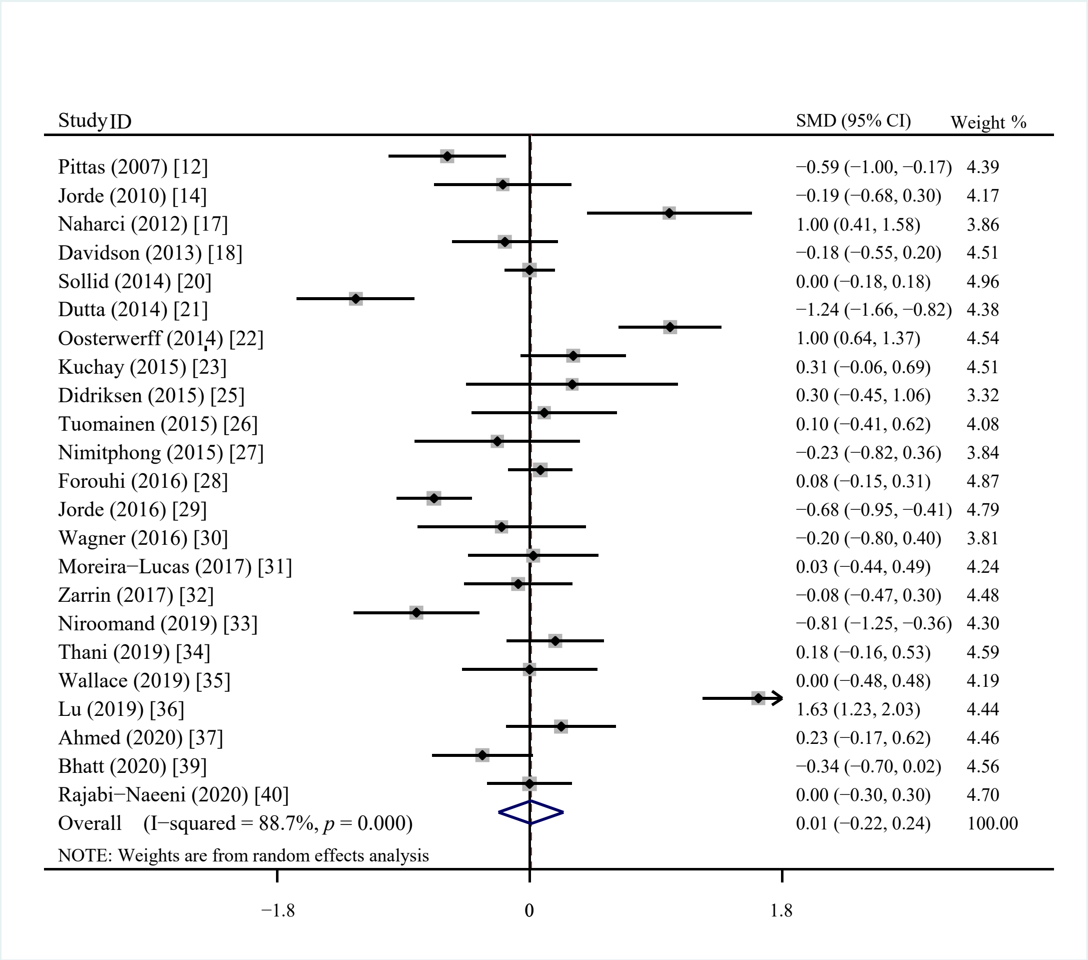

Figure S2. Effect of vitamin D supplementation on body mass index in prediabetes.

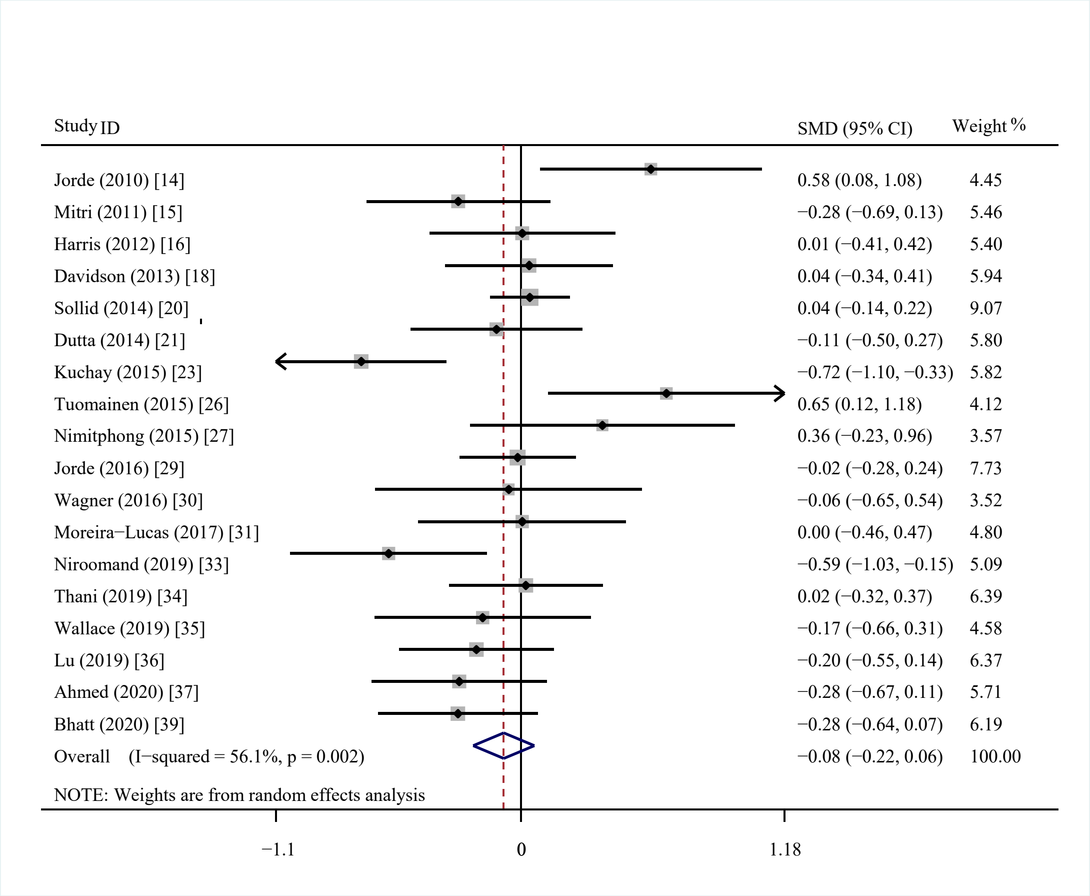

Figure S3. Effect of vitamin D supplementation on 2h oral glucose tolerance test plasma glucose in prediabetes.

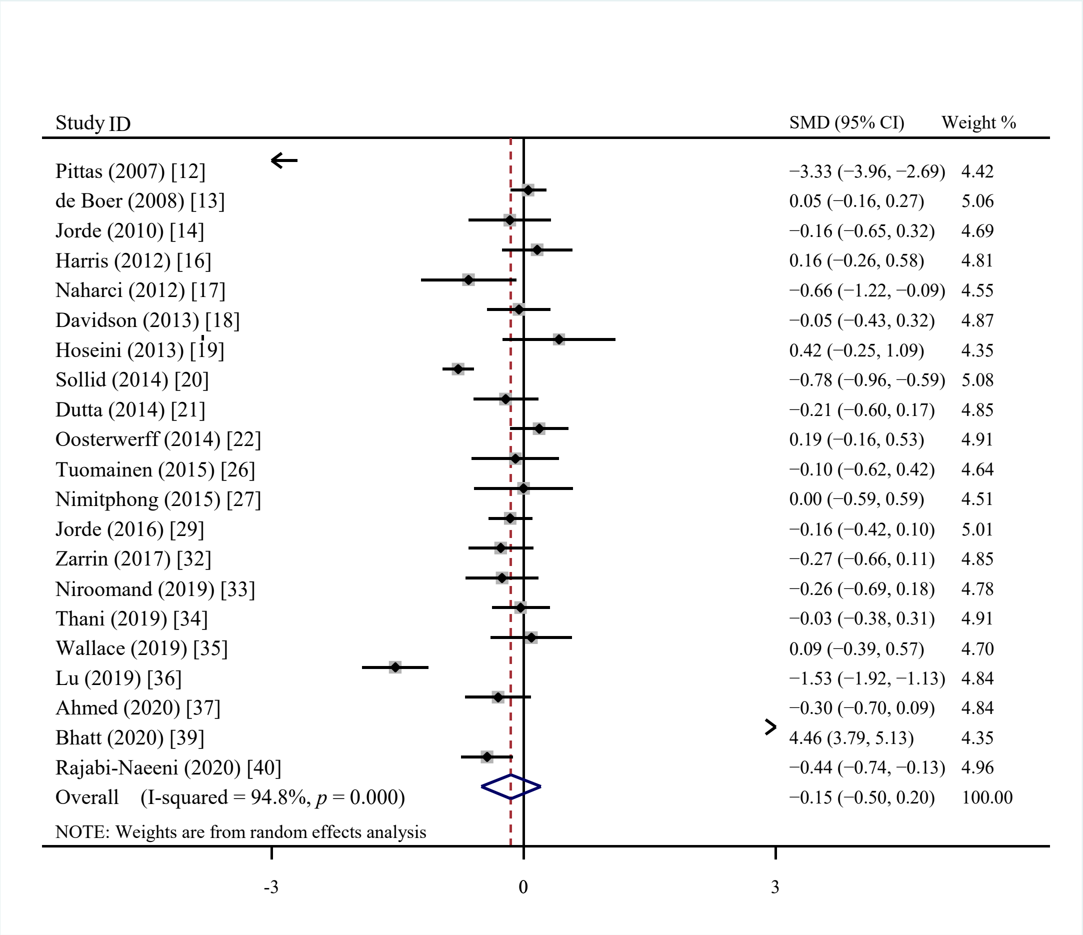

Figure S4. Effect of vitamin D supplementation on insulin resistance by homeostasis model assessment in prediabetes.

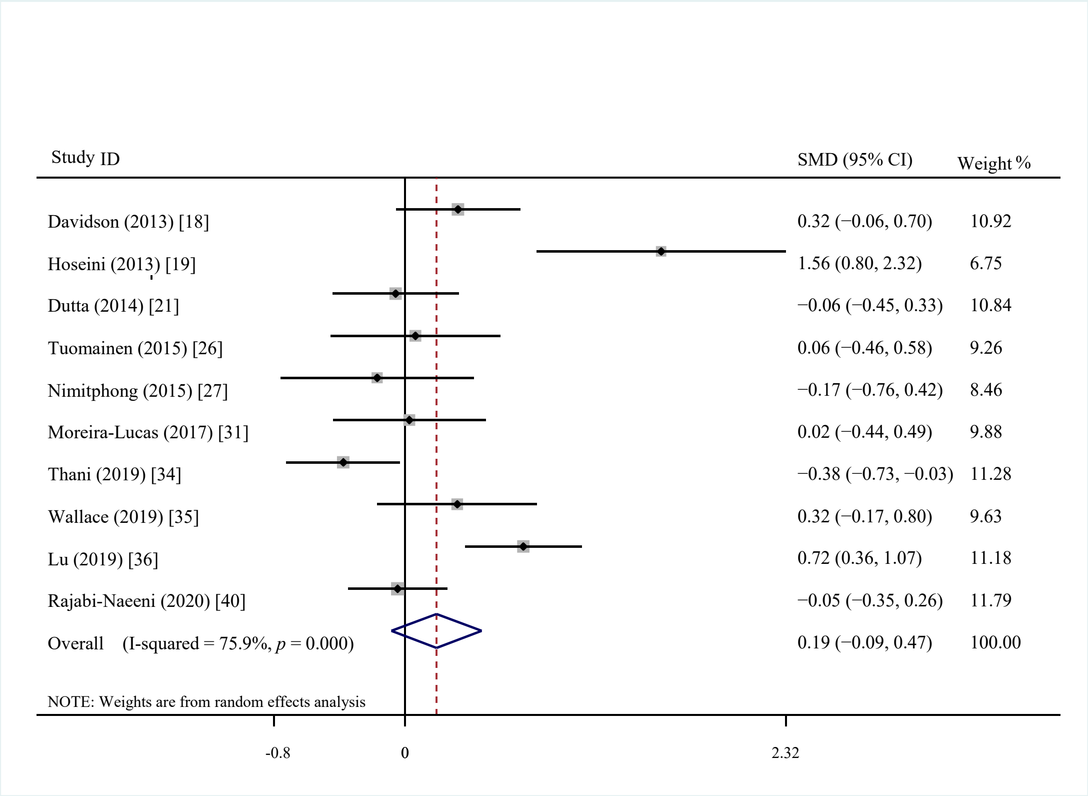

Figure S5. Effect of vitamin D supplementation on homeostasis model assessment of β−cell function in prediabetes.
